# All-Optical Reinforcement Learning In Solitonic X-Junctions

**DOI:** 10.1038/s41598-018-24084-w

**Published:** 2018-04-09

**Authors:** M. Alonzo, D. Moscatelli, L. Bastiani, A. Belardini, C. Soci, E. Fazio

**Affiliations:** 1 Department of Fundamental and Applied Sciences for Engineering, Sapienza Università di Roma, via Scarpa 16, 00161 Roma, Italy; 2 Centre for Disruptive Photonic Technologies, Nanyang Technological University, 21 Nanyang Link, 637371 Singapore, Singapore

## Abstract

Ethology has shown that animal groups or colonies can perform complex calculation distributing simple decision-making processes to the group members. For example ant colonies can optimize the trajectories towards the food by performing both a reinforcement (or a cancellation) of the pheromone traces and a switch from one path to another with stronger pheromone. Such ant’s processes can be implemented in a photonic hardware to reproduce stigmergic signal processing. We present innovative, completely integrated X-junctions realized using solitonic waveguides which can provide both ant’s decision-making processes. The proposed X-junctions can switch from symmetric (50/50) to asymmetric behaviors (80/20) using optical feedbacks, vanishing unused output channels or reinforcing the used ones.

## Introduction

The expression “*reinforcement learning*” is commonly used in computer science to describe those algorithms “of machine learning inspired by behaviorist psychology, concerned with how software agents ought to take *actions* in an *environment* so as to maximize some notion of cumulative *reward*”^1^.

Reinforcement learning regards neural networks or artificial intelligence protocols that self-set by reinforcing a specific information identified by a feedback in the system, in order to solve complex problems. This procedure was inspired by nature, which adopted the stigmergy to transfer information in decentralized systems, thus realizing distributed cognitive processes through many small simple elaborations. The term stigmergy describes precisely that form of communication based on the modification of the surrounding environment^2–5^. Unfortunately, stigmergic software protocols need solution times that increase exponentially with the size of the problem; after many years of research, no improved algorithm has been found to solve these problems within a polynomial time using a deterministic Turing machine. For this reason hardware approaches have been proposed in the past^6–11^; among all, optical solutions to supercomputing seem to win for versatility^12^, in terms of increased fan-in and fan-out, energy-consumption and recursive pre-processing. However, the proposed optical solutions^13–19^ neither reduce the complexity of the problem nor offer technologically efficient procedures without exponentially increasing the demand of physical resources^20,21^.

**Figure 1:**
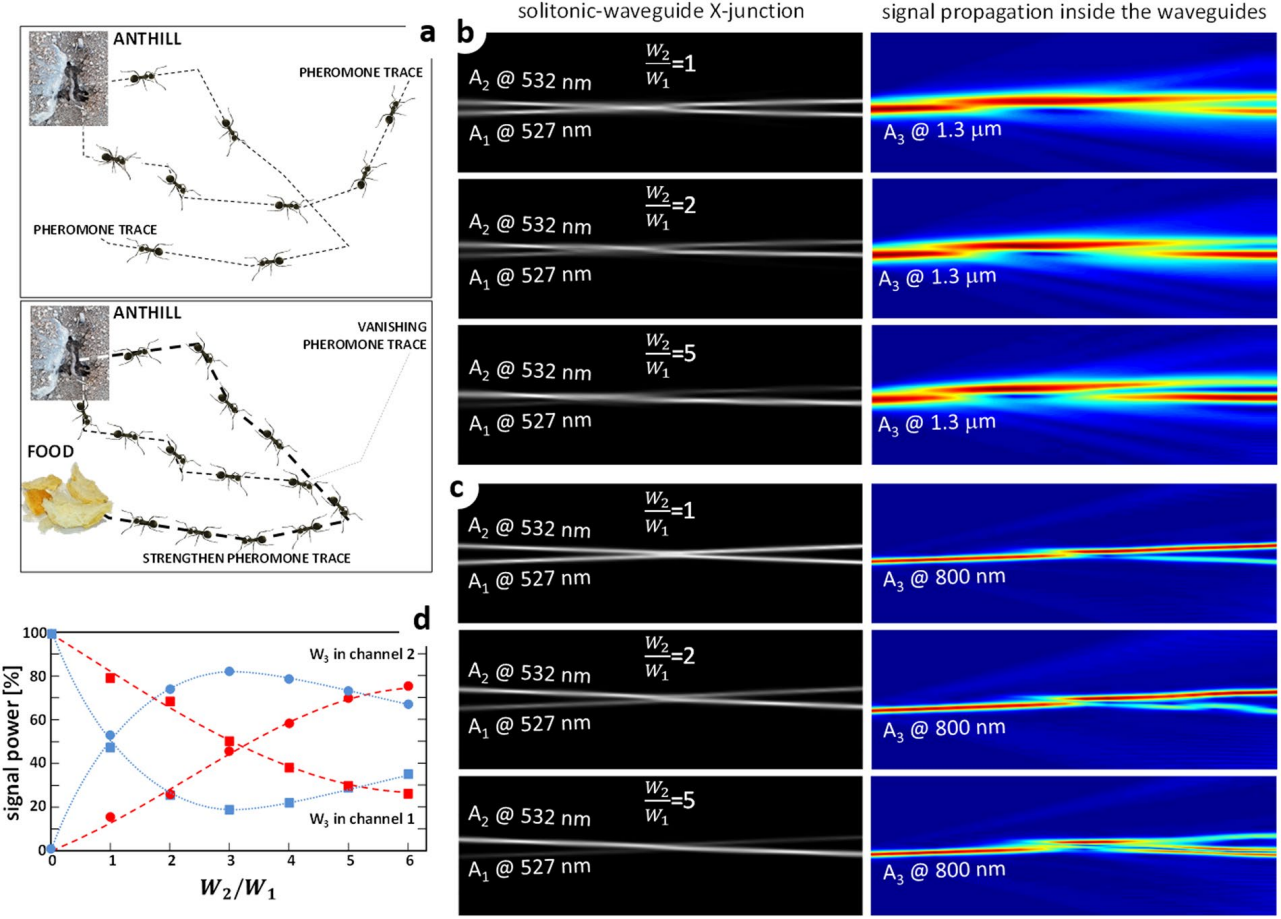
(**a**) Scheme of the Ant Colony procedure for food search. Ants go abroad following random trajectories and leaving a pheromone trace along the path. When food is found, the good trajectory is reinforced by means of the pheromone. All other ants will follow the reinforced path, leaving the other pheromone signals to vanish. (**b**) Simulations of the soliton waveguide formation (black/white on the left-hand side) and of the propagation inside the X-junction of a further signal at 1.3 μm (colored on the right-hand side); (**c**) simulations of the soliton waveguide formation (black/white on the left-hand side) and of the propagation inside the X-junction of a further signal at 800 nm (colored on the right-hand side); (**d)** signal powers at the output. In red the outputs at 800 nm, while in blue the 1.3 μm ones. Circles correspond to the signal power at the exit 2 while the squares correspond to the signal power at the exit 1.

The typical ethologic example of stigmergy performing a reinforcement learning is represented by an ant colony searching for food (Fig. 1a). When ants go looking for food, each one follows a random path, highlighted by the emission of a pheromone trace. When food is found, the right path is reinforced with pheromone on the way back to the anthill. Each other ant crossing such strengthened trace recognizes it as the one to the food and starts following it, reinforcing it as well. After some time, all the other pheromone traces vanish, leaving active just the boosted ones. Is it possible to reproduce the ant’s decisional procedure in a photonic system? Yes: it requires the possibility to modify the surrounding environment, which means adopting a nonlinear medium as host material.

The processing capacity of the ant’s colony is based on two simple decision-making processes: the first concerns the followed trajectory, which can be reinforced or annihilated according to a precise external feedback (which, for the ants, is represented by the quantity of deposited pheromone); the second concerns the chosen direction in a node point, that is at the intersection of two trajectories (the ants will channel into the trajectory with a more intense pheromone trace).

Both processes imply nonlinear responses to the feedback, that can be implemented as hardware photonic elements by using solitonic waveguides. A spatial soliton is a light beam that modifies the refractive index of the host material in order to compensate the natural diffraction^22,23^, getting a self-confined propagation. The material modification induced by solitons can be used as a waveguide to drive other signals inside, exactly like a pheromone trace drives ants along a specific direction. Since the 80’s^24,25^ there has been a growing interest in the waveguiding properties of solitonic beams; among all, the photorefractive solitons represent the most practical ones because of the ultralow light power required for writing^26,27^ waveguides^28^, interconnections^29^, sensors^30^ and other devices.

In 2017 E. Fazio *et al*. ii^31^ have suggested to implement stigmergic systems using such waveguides. In fact, solitonic waveguides can be reinforced (in term of refractive index contrast) or erased according to the intensity of the writing soliton beam, providing the direct hardware solution to the decision-making processes of the ant’s colony.

In the present work, we have theoretically and experimentally implemented both decision-making processes of food research in the hardware photonic way using solitonic channels: in fact, we have reinforced or erased waveguides as well as we have addressed information from one channel to the other of a X junction by using an optical feedback.

## Photonic ant-colony X-junction: numerical simulations

The proposed geometry mimics the decision processes of single ants during their searching for food. Two self-confined beams are generated by visible light beams; the induced refractive index modulations realize two waveguides that can be used by an IR signal. These waveguides cross one each other, realizing a X-junction; each of them simulates an ant trajectory, inside which information can be transported by means of the IR signal. The pheromone signature is given by the writing power of the soliton waveguide that influences the refractive index contrast: the higher is the writing power the larger is the refractive contrast of the related waveguide. Thus playing with the writing intensity, the junction might be symmetrical or asymmetrical, forcing the light to follow different output trajectories. Switching of the transported signal beam from one soliton waveguide to the other is expected. Such configuration was already investigated analytically by Akhmediev and Ankiewicz^32^ for a pure Kerr nonlinearity finding a phase-independent switching between channels, as we have experimented here too.

We have numerically simulated the solitonic-waveguide X-junction using a well-tested FDTD numerical code^30–36^ and monitored the energy transfer at the IR signal wavelength between channels according to the soliton writing power.

Calling *A*_1_ and *A*_2_ the light beams writing the waveguides and *A*_3_ the signal beam, their nonlinear propagations are described by the following set of Helmholtz equations for a saturable nonlinearity:

1 $${\nabla }^{2}{A}_{i}=-\frac{{{\epsilon }}_{NL}{E}_{bias}}{1+\frac{{|{A}_{1}|}^{2}+{|{A}_{2}|}^{2}}{{|{A}_{sat}|}^{2}}}{A}_{i}$$

where *ϵ*_*NL*_ is the nonlinear dielectric constant, *E*_*bias*_ is an external electrical bias necessary for photorefractive screening solitons^30^ and |*A*_*sat*_|^2^ is the saturation intensity. We considered the *A*_1_ beam at 527 nm and the *A*_2_ beam at 532 nm in order to influence the material nonlinearity; the *A*_3_ was instead set at 1.3 μm (the second telecom window), because it does not intervene in the nonlinearity, being just the transported passive signal; we have considered hyperbolic secant transverse profiles for the beams, propagating at a relative angle of 0.4°.

The *A*_3_ signal beam was injected inside the *A*_1_ channel, as shown in Fig. 1b. Like for the pheromone trace, the reinforcement was performed increasing the writing power of channel 2 (where no signal was injected) with respect to the other and monitoring the signal transfer accordingly.

At power ratio $\frac{{W}_{2}}{{W}_{1}}=1$, almost 50% of the propagated signal power remains in the original channel 1 and 50% is coupled into channel 2, giving rise to an almost perfectly balanced 50/50 junction. For $\frac{{W}_{2}}{{W}_{1}} > 1$, the junction is strongly asymmetric, allowing a larger energy-transfer between solitonic waveguides (Fig. 1d, blue marks and lines): the largest one was reached around $\frac{{W}_{2}}{{W}_{1}}=3$, for which almost 80% of the signal energy is switched from the original channel 1 into the crossing and reinforced channel 2. For larger ratios, the coupling does not improve anymore; instead a reversed coupling makes the original channel 1 be pumped again. Consequently the switching stabilizes on a lower efficiency. Such phenomenon was already observed in X junctions with Kerr nonlinearity^37^, where 2-photon and 3-photon absorptions were considered. Here, we do not expect to get any nonlinear absorption, which anyway was not included into the numerical simulations even if the phenomenon was reproduced. We believe that it is a consequence of the signal beam dynamics, as shown in Fig. 1b. After injection inside channel 1, *A*_3_ propagates as TEM00 waveguide mode until the junction is reached. Here, the signal gets a transversal acceleration towards the other waveguide, until the light reaches the opposite wall, on which it is totally reflected (elastic rebound) towards the center. According to the refractive depth of the waveguide crossing point, such rebound can be towards the opposite exit 2 (for low ratios) or again towards the exit 1 (when the high ratios enlarge the transverse dimension of the central area of the junction).

We have also analyzed the possibility to use shorter signal wavelengths: for this purpose we have chosen 800 nm, which nevertheless ensured an insensitivity of the nonlinearity and thus a neutral, passive behavior in propagation within the solitonic waveguides (Fig. 1c). The power transfer from channel 1 to channel 2 is now less efficient, as shown in Fig. 1d (red marks and lines). For power ratios below 3 the signal mainly remained within channel 1, with a very little coupling into the crossing waveguide. Above $\frac{{W}_{2}}{{W}_{1}}=3$, the signal switching becomes more and more efficient, arriving up to 75% for $\frac{{W}_{2}}{{W}_{1}}=6$. A mode-modification is associated to the energy transfer: at power ratio 1, the IR signal coupled within channel 2 maintains a TEM00 bell-shape; at power ratio 2, the coupled signal beam looks swinging inside channel 2 (Fig. 1b). Al larger ratios, the swinging stabilizes in a TEM10 mode (two-lobes) instead of a single-lobe TEM00 mode. Such effect is the consequence of two effects: the saturable nature of the nonlinearity squares the waveguide refractive-index profile (which tends to become a flat-top one increasing the writing power^38^; the angled coupling promotes the excitation of higher modes if available. The mode modification was not observable at 1.3 μm due to the much longer wavelength.

### Waveguide formation

The experimental analysis of soliton X-junction performing reinforcement learning has been realized in lithium niobate crystals using the pyroliton^39^ configuration. Lithium niobate is not the best nonlinear medium for this purpose, in term of speed, because soliton formation takes place in several minutes^30^: however, it has the big advantage of a fine control on the formation process. All measurements have been performed using Z-cut, congruent, striation-free lithium-niobate crystals (of dimensions 12×12×0.5 mm^3^). The necessary electric bias for bright screening solitons was obtained by means of the pyroelectric effect: generating temperature gradients as high as 10°−30 °C between the (0,0,−1) and the (0,0,1) faces (the 12×12 mm^2^ ones), pyroelectric biases of the order of 25–40 kV/cm were efficiently induced. A laser beam at 527 nm (channel 1) was focused onto the (1,0,0) face and propagated along the <1,0,0> direction for about 10 diffraction lengths; it was injected orthogonally to the (1,0,0) face.

**Figure 2:**
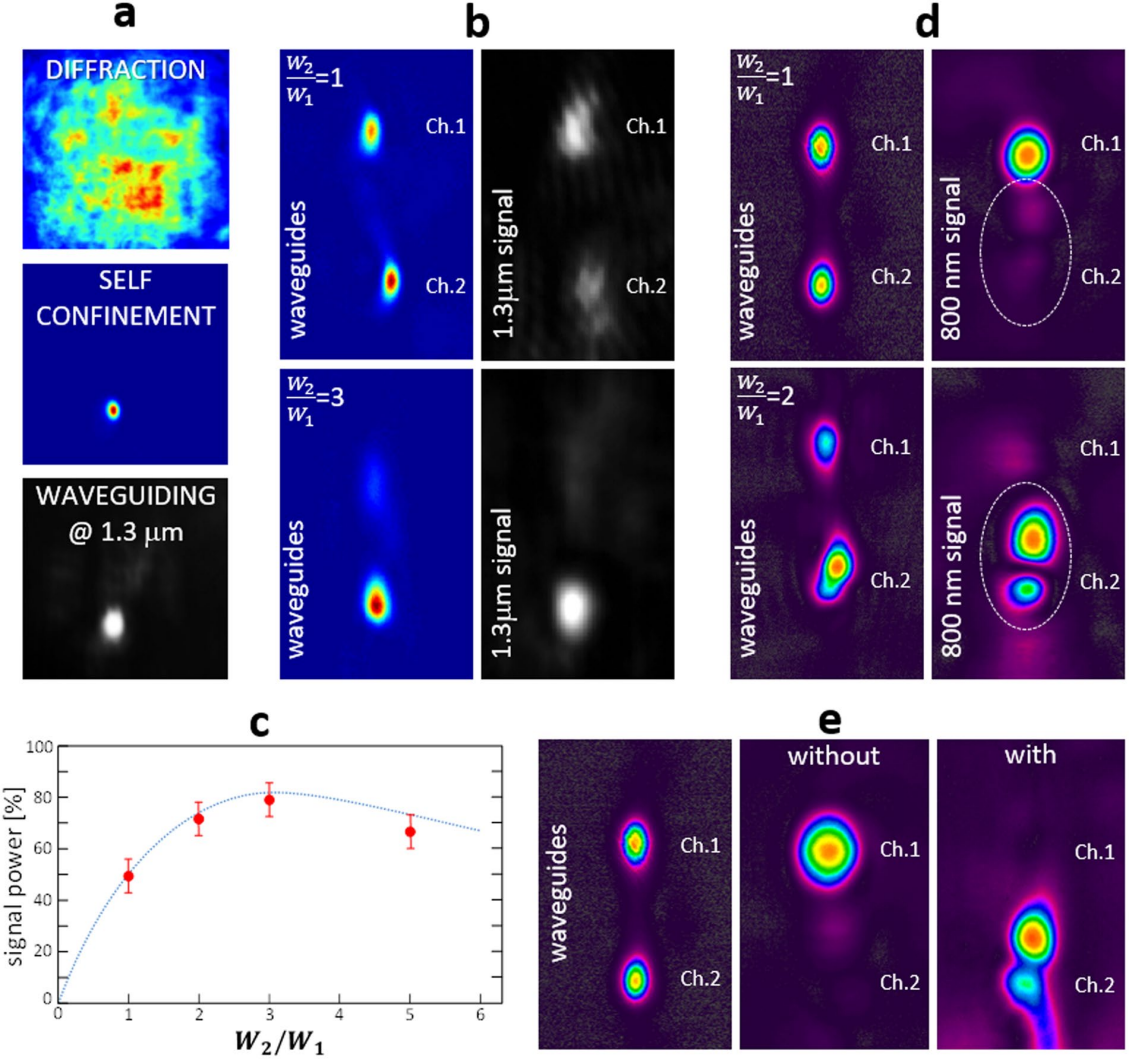
(**a**) Experimental images of the single channel self-confinement and waveguiding; (**b**) experimental transverse profiles of the output solitonic channels (colored images on the left-hand side) and the corresponding transported IR signal at 1.3 mm; (**c**) experimental data and numerical line of the signal power transferred to channel 2 from channel 1; (**d**) experimental transverse profiles of the output solitonic channels (on the left-hand side) and the corresponding transported IR signal at 800 nm; (**e**) experimental transverse profiles of the output solitonic channels (on the left-hand side) and the corresponding transported IR signal at 800 nm for a back-reinforcement.

A single soliton (channel 1) was efficiently generated with 20 μW of power within 4 minutes (see Fig. 2a), reaching a final hyperbolic waist^30,40^ of 7 μm (similar to the input one). Its waveguiding behavior was tested injecting inside it a signal beam at 1.3 μm. Lithium niobate is not photorefractively efficient in the IR: in such a way this beam results completely passive and cannot experiment any confinement unless it is coupled within a waveguide. As shown in Fig. 2a, the IR beam is perfectly waveguided within the soliton channel 1. The propagated mode is larger than the green soliton profile, scaling almost linearly with the wavelength: the hyperbolic secant waist of the soliton at 527 nm was of the order of 7 μm, while the 1.3μm mode had an hyperbolic waist of about 12 μm.

### Co-directional reinforcing

A further beam at 532 nm was injected at an internal angle of 0.4° with respect to the normal direction, and a slight lateral displacement along the [0,1,0] direction in order to ensure a mutual crossing of the beams almost at the center of the crystal. Such beam forms the channel 2 of the X-junction, where no signal is injected at the input. The symmetry or asymmetry of the junction is driven by the writing power of this second solitonic waveguide with respect to the other one. At power ratio $\frac{{W}_{2}}{{W}_{1}}=1$ (Fig. 2b), the transfer is about 50%, making the solitonic X-junction almost perfectly balanced among channels. If the writing power ratio is increased $(\frac{{W}_{2}}{{W}_{1}}=2)$, the junction turns asymmetrical and the propagated signal switches towards channel 2. The largest power transfer was observed at $\frac{{W}_{2}}{{W}_{1}}=3$ as shown in Fig. 2c where a comparison between the experimental and numerical results is reported.

The mode modification during the channel switching was monitored substituting the 1.3 μm IR signal beam with an 800 nm one, as shown in Fig. 2d. In this case both cameras were replaced by a SPIRICON SP620U whose dedicated software allowed to perform beam analysis in real time. At $\frac{{W}_{2}}{{W}_{1}}=1$, the signal still propagates within the original channel 1, with a very low transfer of power into the crossing channel 2. However increasing the power ratio up to $\frac{{W}_{2}}{{W}_{1}}=2$, the signal is switched towards the reinforced waveguide, taking a TEM10 mode shape (twin-lobes distribution) as predicted by the simulations.

### Back reinforcement

Is it possible to induce an asymmetrization of the junction using a feedback from the output gate, as ants do going back to the nest as soon as food is found? We have considered the reinforcement of channel 2 by re-injecting inside it the green writing light from the output. About 70% of the power in channel 2 has been reflected back and re-coupled inside it in contra-propagation. Without reinforcement (Fig. 2e), the signal beam exits from channel 1; with reinforcement, the signal is switched to channel 2, showing in this case too the mode conversion from TEM00 to TEM10. After switching, the original channel 1 remains almost completely depleted, with a really negligible amount of residual signal power.

## Methods

### Numerical simulations

Starting from eq. 1 here reported:

Where A_i_ are the amplitude of the electric fields of the propagating beams, as described before, we use the slowly variable envelope approximation, valid for paraxial beams propagating mainly in the z direction^41^:

2 $$|2{\rm{jk}}\frac{\partial {{\rm{A}}}_{{\rm{i}}}}{\partial {\rm{z}}}|\gg |\frac{{\partial }^{2}{{\rm{A}}}_{{\rm{i}}}}{\partial {{\rm{z}}}^{2}}|$$

and we derive the following equation of propagation for the beams:

3 $$2{\rm{jk}}\frac{\partial {{\rm{A}}}_{{\rm{i}}}}{\partial {\rm{z}}}+\frac{{\partial }^{2}{{\rm{A}}}_{{\rm{i}}}}{\partial {{\rm{x}}}^{2}}+\frac{{\partial }^{2}{{\rm{A}}}_{{\rm{i}}}}{\partial {{\rm{y}}}^{2}}=-\frac{\in {}_{{\rm{NL}}}{{\rm{E}}}_{{\rm{bias}}}}{1+\frac{{|{{\rm{A}}}_{1}|}^{2}+{|{{\rm{A}}}_{2}|}^{2}}{{|{{\rm{A}}}_{{\rm{sat}}}|}^{2}}}\cdot {{\rm{A}}}_{{\rm{i}}}$$

Eq. 3 can be solved by using the finite difference method: let’s consider the generic differential equation (for sake of simplicity in (1 + 1)D configuration)

4 $${\rm{y}}^{\prime} ({\rm{x}})={\rm{f}}({\rm{x}},{\rm{y}}({\rm{x}}))$$

where ${\rm{y}}({\rm{x}})\in {{\rm{C}}}^{2}({\rm{I}})$ and ${\rm{I}}=[{{\rm{x}}}_{0},{{\rm{x}}}_{0}+{\rm{\beta }}]$ is the definition interval. Using the first order Taylor approximation, at the discrete set of points

5 $${{\rm{x}}}_{\ell }={{\rm{x}}}_{0}+\ell \frac{{\rm{\beta }}}{{\rm{n}}}={{\rm{x}}}_{0}+\ell h\,,\,\ell =0,1,\cdots ,{\rm{n}}{\rm{.}}$$

The solution can be written as

6 $${{\rm{y}}}_{\ell +1}\cong {{\rm{y}}}_{\ell }+{\rm{hf}}({{\rm{x}}}_{\ell },{{\rm{y}}}_{\ell })={{\rm{y}}}_{\ell }+h{\rm{y}}^{\prime} ({{\rm{x}}}_{\ell }).$$

With these considerations, eq. 3 can be written as:

7 $$\begin{array}{rcl}{{\rm{A}}}_{{\rm{i}}}({\rm{x}},{\rm{y}},{{\rm{z}}}_{\ell +1}) & = & {{\rm{A}}}_{{\rm{i}}}({\rm{x}},{\rm{y}},{{\rm{z}}}_{\ell })\\  &  & +[\frac{{{\rm{A}}}_{{\rm{i}}}({\rm{x}}+{\rm{h}},{\rm{y}},{{\rm{z}}}_{\ell })-2{{\rm{A}}}_{{\rm{i}}}({\rm{x}},{\rm{y}},{{\rm{z}}}_{\ell })+{{\rm{A}}}_{{\rm{i}}}({\rm{x}}-{\rm{h}},{\rm{y}},{{\rm{z}}}_{\ell })}{{\rm{h}}\cdot {\rm{h}}}\\  &  & +\frac{{{\rm{A}}}_{{\rm{i}}}({\rm{x}},{\rm{y}}+{\rm{h}},{{\rm{z}}}_{\ell })-2{{\rm{A}}}_{{\rm{i}}}({\rm{x}},{\rm{y}},{{\rm{z}}}_{\ell })+{{\rm{A}}}_{{\rm{i}}}({\rm{x}},{\rm{y}}-{\rm{h}},{{\rm{z}}}_{\ell })}{{\rm{h}}\cdot {\rm{h}}}+\frac{\in {}_{{\rm{NL}}}{{\rm{E}}}_{{\rm{bias}}}}{1+\frac{{|{{\rm{A}}}_{1}|}^{2}+{|{{\rm{A}}}_{2}|}^{2}}{{|{{\rm{A}}}_{{\rm{sat}}}|}^{2}}}\cdot {{\rm{A}}}_{{\rm{i}}}({\rm{x}},{\rm{y}},{{\rm{z}}}_{\ell })]\end{array}$$

The input beam profile at the entrance of the crystal is externally provided: we have considered hyperbolic secant beam shapes to simulate the self-confined propagation. In order to avoid numerical instabilities and keep the algorithm convergence under control, the integral of the overall intensity function was calculated and monitored at each simulation step:

8 $${{\rm{P}}}_{{{\rm{z}}}_{\ell }}=\sum _{{\rm{x}},{\rm{y}}}{|{{\rm{A}}}_{1}({\rm{x}},{\rm{y}},{{\rm{z}}}_{\ell })|}^{2}+{|{{\rm{A}}}_{2}({\rm{x}},{\rm{y}},{{\rm{z}}}_{\ell })|}^{2}+{|{{\rm{A}}}_{3}({\rm{x}},{\rm{y}},{{\rm{z}}}_{\ell })|}^{2}.$$

This integral is proportional to the total power carried by the beam and theoretically should remain constant. Numerical approximations and truncations introduce a constant loss that might influences the nonlinear propagation. Such loss, at the distance ${{\rm{z}}}_{\ell }$, was calculated by the error relationship

9 $$ {\mathcal E} {{\rm{z}}}_{\ell }=|\frac{{{\rm{P}}}_{{{\rm{z}}}_{\ell }}-{{\rm{P}}}_{0}}{{{\rm{P}}}_{0}}|$$

where ${{\rm{P}}}_{0}={{\rm{P}}}_{{{\rm{z}}}_{0}}$ is the power at the input surface inside the crystal. The numerical code allows losses lower than 5% over the whole propagation distance.

### Experimental procedure for soliton waveguide formation

The experiments were performed in lithium niobate striation-free single crystals with single-ferroelectric domain polarisation. The external electric bias for obtaining bright screening solitons was internally generated by the pyroelectric effect, inducing a temperature gradient along the c axis, i.e. between the (0,0,−1) and the (0,0,1) crystallographic faces. In such a way, pyroelectric biases of the order of 25–40 kV/cm were efficiently induced by 10°−30 °C temperature gradients.

The laser beams were focused onto the (1,0,0) face and letting them propagate along the <1,0,0> direction. The single soliton waveguide experiments were performed using a single laser beam at 527 nm, injected orthogonally to the input face, with an input waist of the order of 8–9 μm. For the two-waveguide experiments (i.e. the junction) a second beam at 532 nm was injected at 0.4° internal tilt with respect to the <1,0,0> direction. A fine adjustment of the lateral displacement between the two beams ensured a mutual crossing in the center of the crystals. A further laser (at 800 or at 1300 nm) was injected perfectly overlapped with the first 527 laser beam and simulated the transported signals. The switching of such transported beam from one channel to the other was monitored; for this purpose, two different cameras were employed according to the signal wavelength: a Pixelink PL-A741 equipped with a Spiricon SP620U for the visible beams and a Hamamatsu C14041–10U InGaAs for the infrared signal.

## Conclusions

The experimental tests confirm that a soliton-waveguide X-junction can perform efficient reinforcement learning. Starting from a symmetric 50/50 junction realized by two equally intense spatial solitons, any reinforcement of one channel with respect to the other will force the device to asymmetrize. Consequently a switching from 50/50 to unbalanced ratios (70/30–80/20) is induced onto the propagated IR signal. The signal wavelength influences the mode of the switched light, as a consequence of the angled coupling between channels and of the saturating nature of the nonlinearity.

Two important issues are connected with the total losses and with the substrate material. About the first, we have estimated the total losses of the junction to be of the order of 1.5 dB, mainly connected with the coupling. In fact, propagation losses in solitonic waveguides are really low^42^, lower than 0.1 dB/cm; moreover from simulations, the junction would generate losses of the order of 0.4 dB. Experimentally, the losses at the junction point were lower than detectable. Thus, we believe that the total losses are strongly connected with the M^2^ of the beams that influences the quality and homogeneity of the induce self-confined waveguides as well as the light coupling inside. In the present work we have used green beams with ${M}^{2}\approx 1.4$ and a 1.3 μm beam with ${M}^{2}\approx 1.7$; such high values of M^2^ strongly increased the overall losses. Using laser beams with ${M}^{2}\approx 1.1-1.2$ would strongly reduce the total losses, down to a predicted 0.4 dB or better.
